# Quantification of NS1 dengue biomarker in serum *via* optomagnetic nanocluster detection

**DOI:** 10.1038/srep16145

**Published:** 2015-11-05

**Authors:** Paula Antunes, Daniel Watterson, Mattias Parmvi, Robert Burger, Anja Boisen, Paul Young, Matthew A. Cooper, Mikkel F. Hansen, Andrea Ranzoni, Marco Donolato

**Affiliations:** 1 Department of Micro- and Nanotechnology, Technical University of Denmark, DTU Nanotech, Building 345 East, DK-2800 Kgs. Lyngby, Denmark; 2 Insitute for Molecular Bioscience, The University of Queensland, 306 Carmody Road, Brisbane, 4072 QLD, Australia; 3 School of Chemistry and Molecular Biosciences, The University of Queensland, Brisbane, 4072 QLD, Australia

## Abstract

Dengue is a tropical vector-borne disease without cure or vaccine that progressively spreads into regions with temperate climates. Diagnostic tools amenable to resource-limited settings would be highly valuable for epidemiologic control and containment during outbreaks. Here, we present a novel low-cost automated biosensing platform for detection of dengue fever biomarker NS1 and demonstrate it on NS1 spiked in human serum. Magnetic nanoparticles (MNPs) are coated with high-affinity monoclonal antibodies against NS1 *via* bio-orthogonal Cu-free ‘click’ chemistry on an anti-fouling surface molecular architecture. The presence of the target antigen NS1 triggers MNP agglutination and the formation of nanoclusters with rapid kinetics enhanced by external magnetic actuation. The amount and size of the nanoclusters correlate with the target concentration and can be quantified using an optomagnetic readout method. The resulting automated dengue fever assay takes just 8 minutes, requires 6 μL of serum sample and shows a limit of detection of 25 ng/mL with an upper detection range of 20000 ng/mL. The technology holds a great potential to be applied to NS1 detection in patient samples. As the assay is implemented on a low-cost microfluidic disc the platform is suited for further expansion to multiplexed detection of a wide panel of biomarkers.

Dengue fever is one of the major public health concerns in urban and semi-urban areas across tropical and subtropical regions^1^. Over the past decades, it has emerged as the most important mosquito-borne viral disease, spreading globally with a 30-fold increase in incidences. Nowadays, it is endemic in more than 100 countries with half of the world population at risk of infection^2^. The disease is transmitted by infected *Aedes aegypti* mosquitoes and can be divided into four distinct serotypes (DENV 1-4). Undifferentiated symptoms, such as fever, appear rapidly after infection^3^ and it is therefore critical to provide reliable diagnosis in early stages. Viral culture or nucleic acid amplification offer sufficient accuracy and specificity but they are rarely available for decentralized diagnostics in dengue endemic countries^4^. Serological assays are routinely used to confirm potential infections but are, however, less specific^5^. Additionally, the immune response produces immunoglobulins only in later stages of infection and the anamnestic response in secondary infections complicates interpretation of results^6^. Thus, there is a need for sensitive biomarker-based detection technologies offering early and highly specific detection of dengue fever. One of the most established early biomarkers of dengue fever is non-structural protein NS1, released into the bloodstream during viral replication in moderately high concentrations (up to μg/mL levels in acute cases)^7^.

The proven clinical relevance of early NS1 detection has stimulated the development of immuno-chromatographic lateral flow assays^8^, which are rapid immunoassays (15–20 min) designed to provide a non-quantitative readout at the point-of-care (PoC)^9^. However, in many cases the virus serotype and the infection status of patients limit the sensitivity and reliability of these tests^10^ and laboratory confirmation is often required^11^. Enzyme-linked immunosorbent assays (ELISAs) remain the gold standard in dengue endemic areas but the test can take several hours and requires specialized personnel and laboratory facilities^12^.

In response to these challenges several groups have proposed biosensor technologies for NS1 quantification in formats compatible with decentralized diagnostics. Immunosensors based on immunospot assays using fluorescent nanoparticles^13^, surface plasmon resonance^14^, and electrochemical detection^15^,^16^ have recently been presented. These technologies display a growing capacity to provide sensitive NS1 quantification. However, they require multi-step assay strategies and cannot easily be scaled to simultaneous detection of multiple biomarkers. The challenging integration therefore limits their potential for dengue diagnostics^3^.

Here we present a novel optomagnetic lab-on-a-disk technology for NS1 detection based on aggregation of magnetic nanoparticles (MNPs). Previous validation of the readout principle on a model molecular assay in buffer^17^ is now extended to a one-step MNP-based homogeneous immunoassay directly in serum. A biomarker-dependent aggregation of magnetic nanoparticles in raw biological samples is very challenging as nonspecific aggregation cannot be reduced via enhanced stringency of washing steps. Endogenous proteins bind non-specifically and may thus interfere with specific recognition of the target biomarker and impair assay sensitivity. To overcome these challenges, we have designed an anti-fouling surface attachment for the antibodies by means of ‘click’ chemistry^18^. The passivated nanoparticles are deployed in a magnetic agglutination assay, where a few microliters of serum sample are mixed with two identical populations of MNPs functionalized, respectively, with capture (Gus11) and reporter (1H7.4) monoclonal antibodies (mAb) raised against NS1 protein. Sample incubation in a strong magnetic field (hereafter named “magnetic incubation”) induces NS1-mediated MNP aggregation. As a final step, the concentration of the target analyte in solution is quantified by measuring the modulation of the transmitted light upon a magnetic field actuation of the nanoclusters^19^. The entire assay protocol has been implemented on a disc-based platform, which is suited for inclusion of blood-serum separation and for further future expansion to detect a panel of serological markers. We optimize key assay parameters (MNP concentration, incubation conditions, and sample volume) to achieve a clinically relevant NS1 sensitivity range. Ultimately, we present a dose-response curve directly in serum proving robust NS1 quantification in 8 minutes using a serum volume of only 6 μL. The lower limit of detection is established to 25 ng/mL and the sensitivity range of NS1 extends up to 20000 ng/mL.

## Results and Discussion

**Figure 1:**
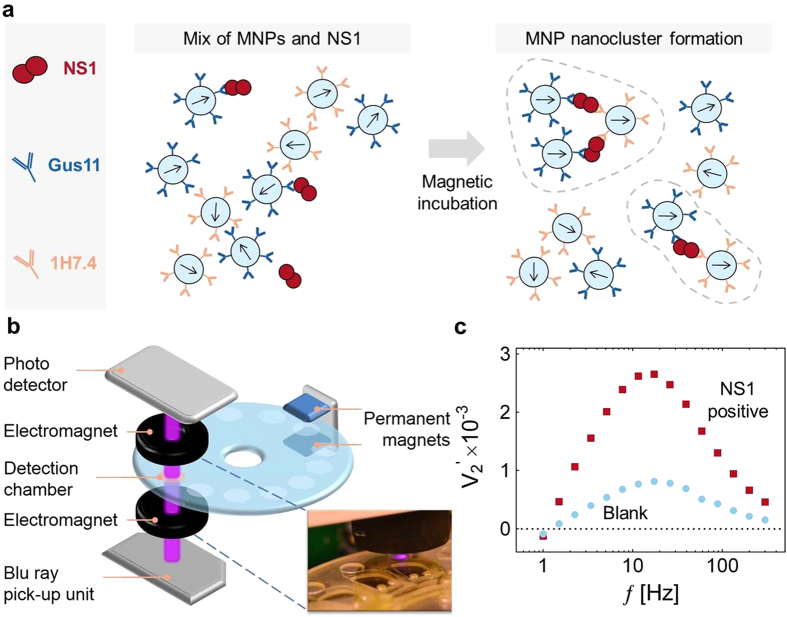
(**a**) Principle of NS1 agglutination assay using functionalized MNPs coated with capture (Gus11) and reporter (1H7.4) monoclonal antibodies. The NS1 target captured by the MNPs (left panel) triggers the formation of MNP nanoclusters during the magnetic incubation (right panel). (**b**) Schematic of readout system consisting of a blue laser light source (from a Blu-ray optical pickup unit, OPU), a photodetector, and two electromagnets that produce the magnetic field modulating the nanocluster rotation and therefore the light transmission. Opposing the detection circuit are placed two permanent magnets to perform the magnetic incubation. (**c**) Spectra of the normalized 2^nd^ harmonic photodetector signal vs. frequency of the magnetic field excitation (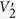 vs. *f* ) for an NS1 negative (blank) and an NS1 positive sample.

MNPs coated with capture (Gus11) and reporter (1H7.4) antibodies bind different epitopes on the NS1 antigen and, for this reason, the presence of NS1 causes linking and agglutination of capture and reporter MNPs with kinetics that can be accelerated by a magnetic incubation step (Fig. 1a). During the magnetic incubation, the strong applied magnetic field causes formation of MNP chains. While in close proximity, the MNPs are subject to Brownian motion, which randomizes their relative orientation and further promotes inter-nanoparticle binding^20^. The formed clusters are subsequently detected by applying an oscillating uniaxial magnetic field along the path of the laser light: the controlled movements of the formed clusters modulates the transmitted light intensity either via a rotation of individual MNPs or nanoclusters with an optical anisotropy, or via a reversible formation of chains of MNPs along the magnetic field. The MNPs used in this work have a negligible remnant magnetic moment and are substantially spherical. Therefore, they do not physically rotate in an oscillating uniaxial magnetic field and there is no link between the induced magnetic moment in the particles and the optical transmissivity. Moreover, in the weak magnetic field applied during the measurements the magnetic dipole interaction between two particles is much smaller than the thermal energy and therefore chain formation due to magnetic dipole interactions is not energetically favorable. Hence, a homogeneous suspension of non-aggregated particles is not expected to produce a modulation of the transmitted light intensity. This agrees well with our observations on uncoated particle suspensions where the particles are kept well separated by electrostatic repulsion (data not shown). However, when particles form dimers or larger agglomerates, there is a linked magnetic and optical anisotropy that produces a modulation of the transmitted light during a cycle of the magnetic field^21^. When the field magnitude is large, the structures tend to align with their long axis along the magnetic field, whereas their orientation becomes random, due to thermal agitation, when the field is low, i.e., the transmitted light intensity peaks twice during a cycle of the magnetic field. Accordingly, the light modulation occurs at 2*f* and the response can be determined from the 2^nd^ harmonic component of the signal measured from the photodetector. We have previously shown that the complex 2^nd^ harmonic signal can be used to extract information related to the real and imaginary magnetic susceptibility of the MNPs and that the in-phase 2^nd^ harmonic signal, 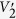, versus frequency of applied field presents a characteristic peak related to the Brownian relaxation frequency of MNP nanoclusters, which is inversely proportional to the hydrodynamic volume of the nanocluster^17^,^22^.

To illustrate the readout method, Fig. 1c shows the in-phase part of the normalized optomagnetic spectra (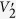 vs. *f* ) for two MNP samples. The blank sample contains MNPs coated with capture and reporter antibodies, respectively, mixed in serum. This sample exhibits a peak at a frequency of *f* = 17 Hz of weak intensity, which is attributed to small population of MNP clusters formed via non-specific interactions during the antibody conjugation and during the magnetic incubation step. The peak position corresponds to a hydrodynamic size compatible with the manufacturer specification^19^. The NS1 positive sample, where NS1 links MNPs with capture and reporter antibodies (cf. Fig. 1a), shows a spectrum with higher intensity and a slight peak shift to low frequencies due to the formation of specific MNP nanoclusters. The number and size of these MNP nanoclusters increase with the NS1 concentration for the investigated range of concentrations to generate signals of increasing intensity. A dose-response curve is extracted by measuring the value of 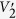 at a frequency of *f* = 17 Hz, corresponding to the position of the peak at low NS1 concentrations.

**Figure 2:**
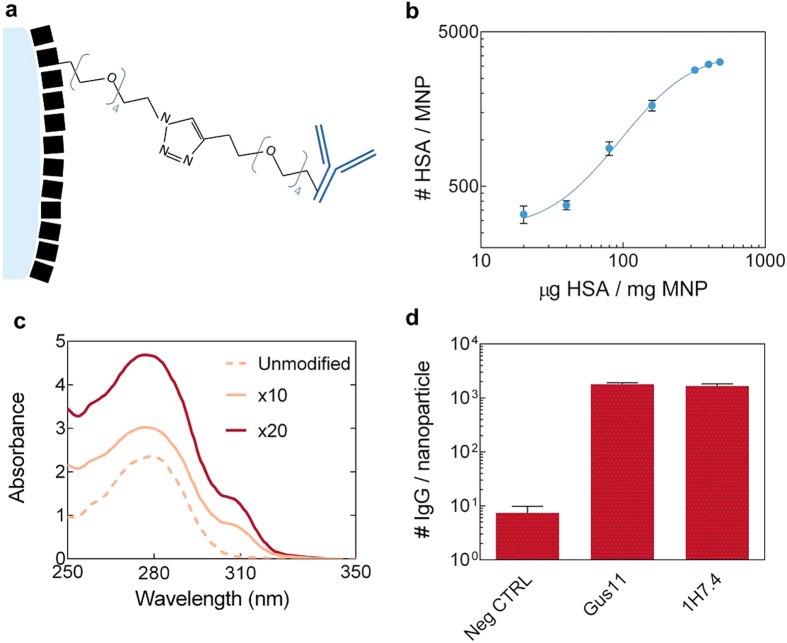
(**a**) Surface molecular architecture: an HSA monolayer (black blocks) on the nanoparticle surface generates a blocking layer onto which bio-orthogonally linked affinity probes are immobilized. (**b**) Number of HSA molecules per MNP as function of the HSA concentration used to identify the amount of HSA to generate a full protein layer on the MNP surface. (**c**) Absorption spectra of Gus11 antibody before modification (light orange dashed curve) and after reaction with a DBCO linker in 10-fold molar excess (light orange curve) and 20-fold molar excess (red curve). The degree of modification is estimated from the characteristic peak at 310 nm. (**d**) Number of immobilized antibodies (Gus11/1H7.4) per MNP estimated using anti-mouse HRP-conjugate antibody assay.

To mitigate the formation of nonspecific aggregates during the magnetic incubation and the influence of complex biological fluids, we have designed an anti-fouling magnetic surface architecture based on generation of a monolayer of blocking proteins (human serum albumin, HSA) on the surface of the nanoparticles. The protein monolayer was used to anchor affinity probes by means of bio-orthogonal Cu-free cycloaddition (Fig. 2a)^23^. We have previously demonstrated that bio-orthogonal conjugation approaches can dramatically impact assay performances in complex matrices^18^. In particular, coating of the hydrophobic regions of polystyrene magnetic nanoparticles with HSA, by means of carbodiimide chemistry helped minimize the adsorption of endogenous interfering agents present in human serum. As it is well known^24^ that the acylisourea intermediate is highly unstable in aqueous conditions, the molar excess of HSA required to fully saturate the carboxyl groups available on the magnetic nanoparticles had to be carefully controlled. Figure 2b shows the measured number of HSA molecules per MNP as function of the mass of HSA used per mass of the MNPs. It is observed that the number of HSA molecules immobilized on the nanoparticle surface progressively increased with the input of HSA. The available surface area was completely occupied by HSA when the reaction occurred in presence of more than 100 μg HSA per mg of nanoparticles. This closely correlates with the value required for a theoretical monolayer^24^. The ligation of HSA molecules on the MNP surface introduced primary amines that were subsequently used as handles for the subsequent layer of the molecular surface architecture. Heterofunctional amine-reactive linkers were covalently bound to HSA to introduce azide moieties on the nanoparticle surface. Similarly, azide-complementary dibenzylcyclooctyne (DBCO) moieties were introduced on the antibodies. The DBCO moiety has a unique spectral fingerprint, with an absorption peak at 310 nm that enabled the degree of modification to be directly monitored by measuring UV absorption of the modified biomolecules. Figure 2c shows the absorption spectra obtained for unmodified Gus11 antibodies and antibodies modified with DBCO linker in 10× and 20× molar excess. We carefully controlled the reaction conditions to ensure to introduce 2-3 DBCO molecules per antibody and verified that the modification did not affect the affinity of the antibody to the NS1 protein^18^. After ‘click’ between azide and DBCO, approximately 2000 antibodies were flexibly linked to the HSA monolayer per particle by means of hydrophilic linkers (Fig. 2d).

**Figure 3:**
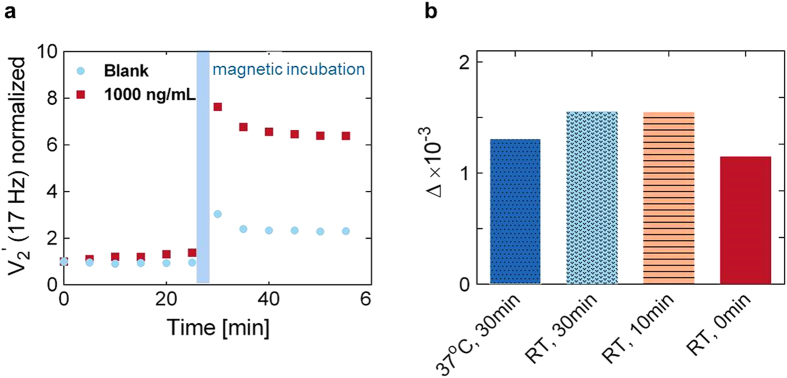
Kinetics of MNP clustering. (**a**) Measurements of 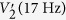 vs. time before and after magnetic incubation for the indicated NS1 concentrations. Values were normalized to the first data point. (**b**) Comparison of Δ-values obtained using NS1 pre-incubation at room temperature (RT) for different times (0 min, 10 min, 30 min) and at 37 °C for a time of 30 min. Δ denotes the difference in 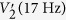 signals after magnetic incubation between the 1000 ng/mL sample and the blank sample.

We first validated the efficacy of the magnetic incubation step by investigating the kinetics of the MNP agglutination in the presence of NS1. A blank sample of serum and a positive sample containing NS1 spiked into serum at a concentration of 1000 ng/mL were used. The MNP concentration was kept constant at 0.1 mg/mL. The reaction was monitored for 60 minutes by measuring optomagnetic spectra every five minutes. The 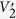 component at *f* = 17 Hz corresponding to the position of the peak for low NS1 concentrations was monitored vs. time *t* for both samples, as shown in Fig. 3a. To eliminate the influence of sample-to-sample variation and to ease graphical comparison, the measurements were normalized by the first measured point (*t* = 0).

The first 30 minutes correspond to the capture phase where NS1 molecules are directly captured onto the surface of the MNPs. This binding reaction triggers the formation of specific MNP nanoclusters and an increase of the intensity of the 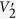 signal, which gives rise to a slope in the 1000 ng/mL sample signal vs. time. The signal increase was moderate in the capture phase since nanocluster formation is diffusion-limited and hindered by electrostatic repulsion between the MNPs. The formation of non-specific clusters in the blank sample (with zero NS1 concentration) takes place on a much longer time scale and in 30 min the signal increase is negligible. Subsequently, the magnetic incubation step was performed for 60 cycles (180 s) to speed up the agglutination kinetics between the MNPs.

The magnetic incubation consisted of two steps: first sample incubation between the two permanent magnets to enhance formation of clusters and then a shaking step to break up non-specific MNP nanoclusters and enable re-orientation of the MNPs. The overall magnetic incubation procedure strongly accelerated the capture dynamics of NS1 as well as the nanocluster formation (Fig. 3a). This result is well in line with previous literature which shows that the application of continuous^25^ or pulsed^21^ magnetic field efficiently enhances MNP agglutination. The data in Fig. 3a show that magnetic incubation provides a 3-fold increase of the signal amplitude in the positive sample compared to the blank sample. Non-specific interactions mediated by magnetically induced collisions, physisorbed biomolecules from the human serum and residual hydrophobic regions (e.g., triazole groups) could occur, even in the presence of a HSA monolayer. We observed a net decrease in the signal amplitude during the subsequent 30 minutes for both the 1000 ng/mL and blank samples. Since this effect is target independent, we attribute the decrease to the loss of weakly bound nanoclusters formed due to non-specific interactions.

To maximize the biomarker depletion during the incubation phase, we have evaluated the assay performance as function of temperature and time. Kinetic theory dictates that increased thermal energy results in higher motility and collision rate between biomolecules in solution. In addition, the antibody association-dissociation constant depends on temperature^26^. We define ∆ as the difference between the 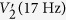 signals for the positive sample and the blank sample just after magnetic incubation. Figure 3b shows ∆ measured for a 1000 ng/mL sample with four different incubation conditions. The MNP concentration, the sample volume and the magnetic incubation protocol were kept constant and the same as in the previous experiment. The results indicate that a higher or similar value of Δ is obtained at RT compared to 37 °C. Moreover, pre-incubation prior to the magnetic incubation is observed to only result in minor signal improvement compared to no pre-incubation. Hence, we can conclude that the NS1 capture and MNP clustering mainly take place during the magnetic incubation. Other experimental conditions, such as a two-step approach with capture and reporter nanoparticles incubating with the sample in successive steps, were shown to provide a lower ∆ (Supplementary Information, Section S2).

**Figure 4:**
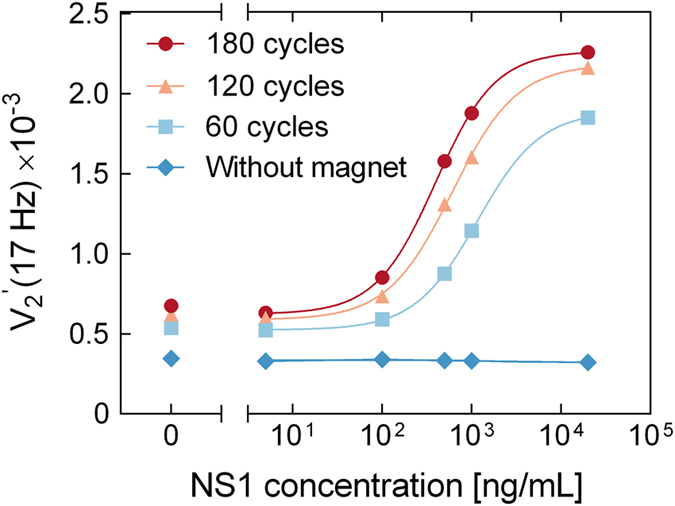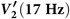 signal vs. NS1 concentration for the indicated number of magnetic incubation cycles (each lasting 3 s in total). Each protocol was applied for the indicated concentrations of NS1 spiked in serum (total sample used 6 μL) and a MNP concentration of 0.1 mg/mL Lines are curve fits obtained using the four parameter logistic model as described in the Supplementary Information, Section S3.

We further investigated the parameters of the magnetic incubation. We found that prolonged exposure to the magnetic field resulted focusing of the MNPs onto one side of the microfluidic chamber due to the field not being completely homogeneous. For a magnetic field exposure time of 1 s, we achieved a dynamic cloud of MNPs perfusing the microfluidic chamber. The shaking step time, acceleration and speed needed to optimally redistribute the particles were optimized empirically. The optimal magnetic incubation protocol length for the NS1 assay was identified by varying the total number of magnetic incubation cycles. Figure 4 shows 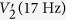 vs. NS1 concentration spiked in serum (total sample volume 6 μL) measured after the indicated number of magnetic incubation cycles. In all cases, MNPs were used at a concentration of 0.1 mg/mL.

Without magnetic incubation, the signal was low and identical for all NS1 concentrations. After magnetic incubation, the signal increased by a factor of 3–10 with a higher increase for higher NS1 concentrations. For all investigated NS1 concentrations, the signal amplitude was higher for the longest magnetic incubation, which causes as well higher signal in the blank sample (0 ng/mL sample). The lines in Fig. 4 are curve fits obtained using the four parameter logistic (4PL) model with the parameters given in Table S1 (Supplementary Information, Section S3). Analysis in terms of the 4PL model supports that 180 cycles of magnetic incubation provides the highest sensitivity. In addition, the highest 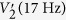 amplitude ratio between the 100 ng/mL sample and the blank after 180 cycles also indicate that this number of cycles results in a lower LOD. To further optimize the assay conditions, the effect of the MNP concentration was studied (Supplementary Information, Section S4) and the optimal MNP concentration was established to 0.1 mg/mL.

**Figure 5:**
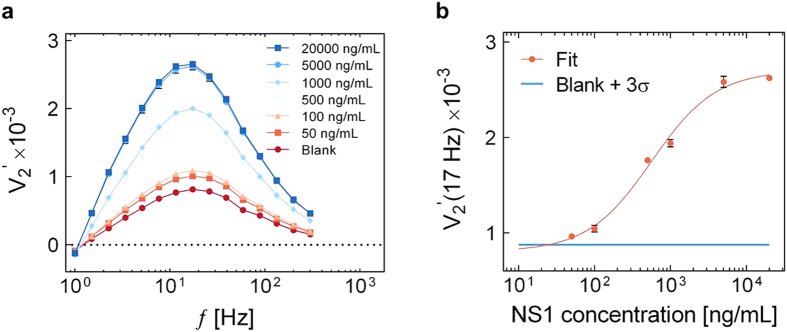
(**a**) 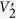 spectra of 0.1 mg/mL MNP suspension mixed with the indicated concentrations of NS1 in serum. Lines are guides to the eye. (**b**) 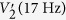 vs. NS1 concentration in serum. Error bars are standard deviations obtained from triplicate experiments. The line is a curve fit obtained using the four parameter logistic model. The horizontal line indicates the blank sample signal plus 3 standard deviations.

The performance of the proposed technology was evaluated using the above optimal assay protocol on NS1 spiked into a serum sample with a total volume of 6 μL. This corresponds to the typical volume that can be extracted out of a single drop of blood from, for example, a patient fingerprick. Figure 5a shows 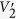 spectra obtained for the indicated NS1 concentrations. The spectra show a clear signal increase for increasing NS1 concentration with a peak exhibiting a slight shift towards lower frequencies for high NS1 concentrations. For the high investigated NS1 concentrations, multiple NS1 molecules are likely to bind to each MNP (an NS1 concentration of 10000 ng/mL corresponds to 500 NS1 molecules per MNP). Accordingly, larger clusters of MNPs are expected to form with a correspondingly larger hydrodynamic size and lower Brownian relaxation frequency.

Figure 5b shows 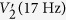 vs. NS1 concentration obtained from triplicate experiments. The solid line shows a curve fit of the 4PL model (Supplementary Information, Section S3) to the data. The Hill coefficient value for this curve was 1.03 and the *EC50* value was 566 ng/mL. The coefficient of variation did not exceed 2% for any of the measured samples. This indicates that the method is also robust in serum. The limit of detection (LOD), calculated using the blank signal plus three standard deviations, was 25 ng/mL. The dose-response curve showed a large dynamic range covering almost three orders of magnitude of NS1 concentrations. Saturation is reached at high concentrations partly due to the fact that large MNPs are spun on the side of the chamber during magnetic incubation, which is confirmed by the absence of further peak shift. If needed, the linear range could be tailored to, e.g., higher concentrations, by tuning the magnetic incubation protocol and the MNP concentration.

The presented assay dynamic range and LOD are comparable to those obtained using other sensing technologies based on MNP clustering^21^,^25^,^27^,^28^,^29^. Thanks to the efficient antibody coverage on the MNPs surface a comparatively larger dynamic range is obtained in comparison to other approaches where magnetic field actuation is used as well to enhance particle agglutination^21^,^29^. A lower limit of detection may potentially be reached in an MNP clustering assay with a miniaturized nuclear magnetic resonance (NMR) based readout^28^,^30^,^31^. However, a simple optical readout, compatible with any optical transparent microfluidic system and which does not require any sophisticated electronic components or complex cartridge manufacturing^32^ holds a stronger potential to impact POC diagnostics of infectious diseases. It is indeed important to put these results into perspective for the detection of *flavivirus* induced diseases, like dengue fever, which predominantly occur in remote areas with minimal access to well-equipped clinical laboratories. This particular diagnostic niche is dominated by ELISA and lateral flow devices due to their cost-effectiveness. ELISA delivers sensitive detection at the expense of a time consuming multi-step procedure whilst the latter offers simplicity with moderate analytical/clinical sensitivity^3^. Our approach delivers a performance comparable to ELISA, but with a much simpler assay protocol and a much shorter total assay time on a lower sample volume. Moreover, the integration with centrifugal microfluidics enables full automation in an out-of-lab setting and is anticipated to lead to future multiplexed detection from the same drop of blood.

The levels of circulating NS1 in dengue infected individuals can vary depending on the severity of the disease, its immunological status (primary or secondary infection) and its serotype. In acute cases, circulating NS1 values in the μg/mL range have been reported^7^. However, values below 10 ng/mL may occur for early stages of the disease^33^. Current ELISA commercial kits, the gold standard for NS1 detection, only partially cover this low range of concentrations and poor clinical sensitivities for DENV-2 and DENV-4 serotypes have been reported^34^. An improved LoD would be beneficial for early diagnosis of dengue. The presented technology has the potential to deliver rapid and fully automated diagnosis in the field. The main factor limiting the current LOD is the nonspecific binding of MNPs. Further optimization of the MNP surface architecture and the assay protocol are likely to reduce the non-specific background. Moreover, more sophisticated analysis methods, for example, taking into account the full measured frequency spectra before and after magnetic incubation rather than the value at a single frequency, may also improve the sensitivity and LOD. These improvements are essential for translation to the clinics and usage in the field and are topics of ongoing work. We are presently developing novel affinity probes capable of cross-reactive interaction across all four dengue serotypes. These will enable estimation of the clinical sensitivity and specificity by testing samples of dengue infecting patients. Precise quantification of other biomarkers, including serological markers, could help triaging patients and may further provide valuable data for surveillance and epidemic control.

In conclusion, we have presented a novel biosensing platform for dengue NS1 detection spiked in human serum. The assay is fully integrated and requires only a few microliters of clinical sample to be mixed with the bioactive nanoparticles. External magnetic fields actuate the nanoparticles in solution, triggering both biomarker-induced nanocluster formation and a time-dependent transmitted light modulation profile, which correlates with the number of nanoclusters in solution. We have demonstrated minimal non-specific nanoparticle aggregation by coating nanoparticles with an anti-fouling surface molecular architecture. Bio-orthogonal ‘Cu-free’ click chemistry enables efficient immobilization of >2000 high affinity antibodies against dengue NS1 protein. The result is a fast (8 minutes) and automated dengue fever diagnostic assay compatible with a fingerprick sample volume. The presented technology shows a limit of detection of 25 ng/mL and a wide dynamic range up to 20000 ng/mL.

## Materials and Methods

### Chemicals and materials

The generation and characterization of 1H7.4 and Gus11 antibodies have been described previously^11^,^14^,^35^,^36^. In brief the antibodies were produced by immunizing BALB/c mice with subcutaneous injections of NS1 protein, regularly boosted with subsequent monthly injections. Blood samples were collected by tail bleed and the antibody titer was checked by ELISA to identify the mice with the strongest immune response prior to culling. The harvested spleen cells were fused with plasmacytoma cell line SP2/0 at a ratio 10:1 and subsequent selection and characterization of the antibodies was performed by ELISA. To generate purified stocks, antibodies were isolated from ascites fluid using affinity chromatography on protein G sepharose (HiTrap Protein G HP, GE Healthcare) as per manufacturer instructions. After elution in 100 mM glycine pH 2.7, purified antibody stocks were buffer exchanged using centrifugal filtration (50kDa MWCO Amicon Ultra-15, Millipore) into phosphate buffer saline (PBS). Protein concentrations were determined using the Bicinchoninic acid (BCA) Protein Assay (Pierce).

Superparamagnetic polystyrene nanoparticles with embedded magnetic nanograins and a nominal diameter of 170 nm and COOH surface were acquired from Merck (NJ, USA). Purified recombinant Dengue Virus NS1 glycoprotein was obtained from Hawaii Biotech (Lot. 2DNS1). All chemicals were purchased from Sigma Aldrich, unless otherwise noted. MNP storage buffer was prepared with PBS and Tween 20. In this investigation, NS1 was always diluted in CRP-free serum from HyTest Ltd. (Turku, Finland).

The methods were carried out in accordance with the approved guidelines. All experimental protocols were approved by the DTU Nanotech work environment committee. The procedure for coupling of Gus11 and 1H7.4 antibodies to separate populations of MNPs is described in the Supplementary Information, Section S1. Unless otherwise stated, all MNP suspensions were 1:1 mixtures of MNPs with Gus11 and 1H7.4 antibodies and stated MNP concentrations refer to the mixture of MNPs.

### Experimental Setup

Figure 1b shows a picture of the readout system previously described in refs 17 and 22. In brief, the major optical elements were a Sanyo Blu-ray optical pick-up unit (λ = 405 nm) and a ThorLabs PDA36A photodetector. The microfluidic disc was placed between two electromagnets used to generate a sinusoidal uniaxial magnetic field parallel to the laser beam direction of fixed amplitude *B*_0_ = 2 mT at a frequency *f* up to 300 Hz. The pre-amplified signal from the photodetector was acquired using a National Instruments 6251 data acquisition (DAQ) card and analyzed through a software-based lock-in amplifier. The same DAQ card controlled a customized circuit, embedded in the optical pickup unit control board, used to provide the alternating current to the electromagnets. The software extracted the intensity of the 2^nd^ harmonic signal from the photodetector and its phase lag with respect to the magnetic field excitation and presented it as the complex 2^nd^ harmonic lock-in signal. The software also extracted the average signal from the photodetector. All signals presented below have been normalized with the average photodetector signal and are hence dimensionless. The normalized in-phase 2^nd^ harmonic photodetector signal is given the symbol 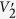.

The disc was connected to a rotary stepper motor (Maxxon Motor, mod. 273756, Switzerland) used to perform the microfluidic operations. The motor was operated using a digital positioning controller (Maxxon, mod. 347717) via a custom-made Labview program (National Instruments, US). A pair of permanent magnets was placed on the radial position opposite to the two electromagnets and was used to provide a strong approximately homogeneous field during the magnetic incubation step. The field measured at the center of the measurement chamber was around 90 mT.

### Disc fabrication

Centrifugal microfluidic discs were manufactured using Poly(methyl methacrylate) (PMMA, Axxicon, Netherlands) and pressure sensitive adhesive (PSA, 90106, Adhesives Research, Ireland) as described by Donolato *et al.*, 2014. Each microfluidic disc was made from three 600 μm thick PMMA DVD halves. Reservoirs were created by cutting through the first PMMA disc using a CO_2_ laser (Mini 18, 30W, Epilog, USA). Inlets were laser machined in the second disc and the third disc was used as support. Alignment holes were also cut in all three discs. Following these steps, fluid channels were created in two PSA sheets using a blade cutter machine (Silhouette, USA). The PSA was laminated on the bottom and top discs, which were then aligned and bonded to the central disc containing the reservoirs. This rapid prototyping process allowed for the complete fabrication of a disc with eighteen microfluidic chambers is less than 20 min.

### Measurement protocol

Experiments were performed as follows: each MNP population was diluted in the proper buffer to the required concentration followed by vortex and ultrasound treatment for 2 min (Model 150 V, Biologics, US). NS1 was spiked in human serum and aliquoted accordingly. Samples were prepared off-disc by mixing the MNP suspension with 6 μL of NS1 sample and immediately transferred into the disc loading chamber. The disc was then placed on the previously described setup and spun (18 Hz, 3 sec) to drive the sample to the detection chamber. The chamber was aligned with the Blu-ray laser and the two electromagnets, and a first optomagnetic spectrum was recorded. An optomagnetic spectrum consisted of a measurement of 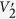 vs. *f* for fifteen logarithmically equidistant values of *f* between 1 and 300 Hz and it was recorded in 60 s. Subsequently, the sample was exposed to a magnetic incubation step. The magnetic incubation protocol comprised a number of cycles, each with 1 sec of incubation between the permanent magnets followed by 2 sec of mixing (one full accelerating revolution in each rotation direction). The first step enhanced magnetic agglutination of the MNPs. The second step promoted mixing of the MNPs, random MNP rotation, and random antibody-antigen encounters to accelerate the recognition^21^. Magnetic incubation protocols with 60, 120 and 180 cycles were investigated. Finally, after magnetic incubation, the chamber was aligned with the laser to record the second optomagnetic spectrum.

## Additional Information

**How to cite this article**: Antunes, P. *et al.* Quantification of NS1 dengue biomarker in serum *via* optomagnetic nanocluster detection. *Sci. Rep.* **5**, 16145; doi: 10.1038/srep16145 (2015).

## Supplementary Material

Supplementary Information
