# Supplementary material for: Quantification of NS1 dengue biomarker in serum via optomagnetic nanocluster detection
Source: Sci Rep. 2015 Nov 5;5:16145. doi: 10.1038/srep16145 (PMC4633614; doi:10.1038/srep16145)
Supplement: Supplementary Information [file srep16145-s1.pdf]

Supplementary Information

Quantification of NS1 dengue biomarker in serum *via* optomagnetic nanocluster detection

Paula Antunes[1], Daniel Watterson[3], Mattias Parmvi[1], Robert Burger[1], Anja Boisen[1], Paul Young[3], Matthew A. Cooper[2], Mikkel F. Hansen[1], Andrea Ranzoni[2], Marco Donolato[1*]*

[1]Department of Micro- and Nanotechnology, Technical University of Denmark,
DTU Nanotech, Building 345 East, DK-2800 Kgs. Lyngby, Denmark

[2]Insitute for Molecular Bioscience, The University of Queensland,
306 Carmody Road, Brisbane, 4072 QLD, Australia

[3]School of Chemistry and Molecular Biosciences, The University of Queensland,
Brisbane, 4072 QLD, Australia

*Address correspondence to marco.donolato@gmail.com, a.ranzoni@imb.uq.edu

S1. Coupling of antibodies to MNPs

Human serum albumin HSA (Sigma Aldrich, A9511) was immobilized on the magnetic nanoparticles by means of carbodiimide chemistry. HSA was dissolved in phosphate buffer saline (PBS) with 0.1% Pluronic F-127 (PBSP). 50 mg of HSA was added to a particle batch of 500 µL at a nanoparticle concentration of 5% (w/v). After overnight incubation, the particles were washed three times and resuspended in 500 µL of PBSP. Particles were tested with Bicinchoninic acid assay (BCA, Thermoscientific 23227) for quantification of the amount of immobilized HSA. 2 µL of particles at 5 % (w/v) were incubated with 200 µL of BCA reagent (diluted as per the manufacturer instructions) and incubated at 37 ^{o}C for 30 minutes. The absorbance at 562 nm relates to the amount of bound surface protein (Fig. S1a). 1.25 mg of NHS-PEG$_4$-N$_3$ (ClickChemistryTools, AZ103-25) linker was added to a batch of 500 µL of HSA coated magnetic nanoparticles and incubated at room temperature for 1 hour. The particles were washed three times with PBSP (final volume 500 µL).

The amount of N$_3$ linker was quantified by means of a fluorescence assay as follows: 5 µL of particles were added to 10 µL of alkyne-FITC at 100 mg/mL (Click chemistry tools, TA106). 100 µg of freshly prepared sodium ascorbate (Sigma Aldrich, 268550) was added to the reaction

together with 0.5 nmol $CuSO_4$ (Sigma Aldrich, 209198) and 2.5 nmol THPTA (Sigma Aldrich, 762342). After 2.5 hours incubation at 37°C the particles were washed three times with PBSP. Fluorescence intensity correlated with a standard curve revealed the density of N_3 groups on the nanoparticle surface (Fig. S1b).

Immunoglobulins (Gus11 and 1H7.4) were buffer exchanged to PBS and a 20-fold molar excess of NHS-PEG_4-DBCO (ClickChemistryTools, A134-10) linker was added to antibodies and allowed to react for 1 hour at room temperature. Click reaction was performed at 37 °C for 3 hours. The particles were washed three times with PBSP (final volume 500 µL). The particles were tested for the presence of antibodies on the surface using anti-mouse IgG-HRP as follows: 5 µL of the particles were incubated with the anti-mouse antibodies (Jackson Immunoresearch 115-035-003, diluted 1:5000) for 1 hour at 37°C. Particles were washed three times with PBSP to remove unbound anti-mouse antibodies. 100 µL of 3,3',5,5'-Tetramethylbenzidine (TMB) reagent was added and left for 15 minutes for color development. 20 µL of 1M H_2SO_4 was added to stop the reaction and measurement was done at 450 nm within 30 minutes after the reaction was stopped. Amount of antibodies was calculated comparing to a standard curve (Fig. S1c).

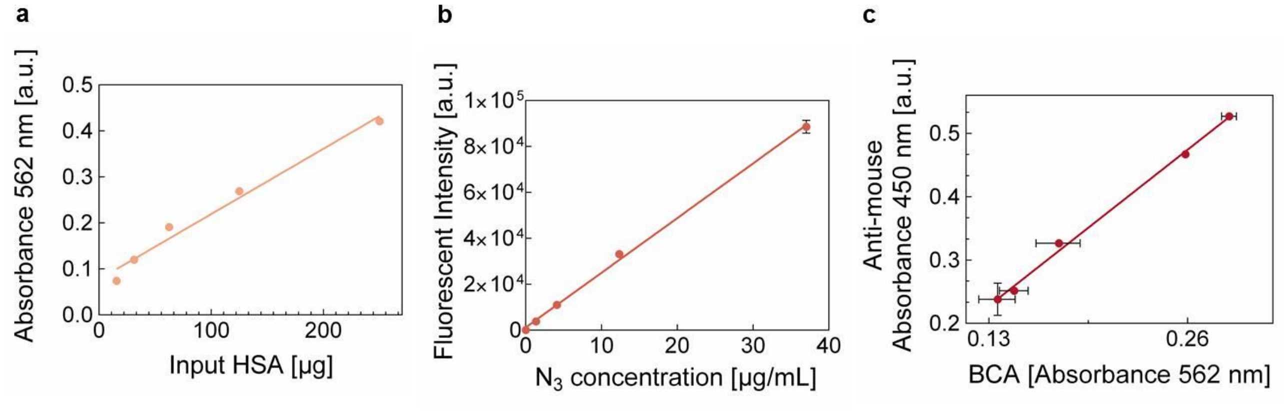

Fig. S1 (a) Calibration curve correlating the amount of HSA in the reaction with the absorbance of BCA assay at 562 nm. (b) Calibration curve correlating the amount of NHS-PEG4N3 on the nanoparticle surface. The density of azide groups is quantified by polar cycloaddition with alkyne-FITC. (c) Calibration curve used to correlate the colorimetric signal obtained with anti-mouse HRP bound to the immobilized immunoglobulins.

S2. Comparison of one- and two-step assay protocols

Direct comparison between one-step and two-step immunoassay protocols was performed. 24 µL of 0.1 mg/mL MNP suspension was incubated with 6 µL of 100 ng/mL or 1000 ng/mL of NS1 diluted

in serum. The order of mixing of functionalized MNPs (first Gus11 or 1H7.4) with the sample, capture phase incubation time (0 or 10 min) and the number of steps (one or two) in the assay were investigated using the following protocols:

- **A.** *1H7.4*: Only 1H7.4 MNPs mixed with NS1. No pre-incubation time.
- **B.** *Gus11*: Only Gus11 MNPs mixed with NS1. No pre-incubation time.
- **C.** *Both 0 min*: 1H7.4 MNPs and Gus11 MNPs mixed with NS1. No pre-incubation time.
- **D.** *Gus11+1H7.4*: Gus11 MNPs mixed with NS1 and incubated for 10 min. After that 1H7.4 MNPs were added.
- **E.** *1H7.4+Gus11*: 1H7.4 MNPs mixed with NS1 and incubated for 10 min. After that Gus11 MNPs were added.
- **F.** *Both 10 min*: 1H7.4 MNPs and Gus11 MNPs mixed with NS1 and pre-incubated for 10 min.

After the above pre-incubation steps, samples were loaded into the disc, magnetic incubation was performed (180 cycles) and the signal was acquired. Figure S2 presents $V_2'(17\,\mathrm{Hz})$ for NS1 concentrations of 100 ng/mL and 1000 ng/mL for the above pre-incubation protocols.

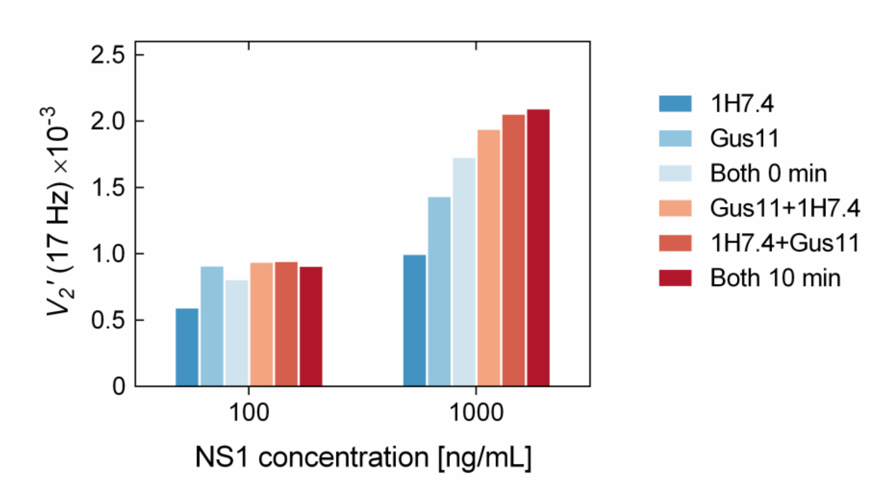

Fig. S2 $V_2'(17\,\mathrm{Hz})$ measured after magnetic incubation for two NS1 concentrations after exposure to the six different pre-incubation protocols described in the text. Blue and red bars indicate results obtained without and with 10 min pre-incubation.

As expected, a higher signal is obtained when both antibodies are used simultaneously compared to when using only one kind of functionalized MNPs. Protocols that include 10 minutes of capture phase incubation (red bars) have slightly higher amplitude than those with 0 min incubation (blue bars). The order of used MNPs did not affected results significantly. Therefore, to achieve a fast but sensitive assay, all experiments were performed mixing both populations of MNPs together with the NS1 sample with no pre-incubation prior to the loading into the disc.

S3. Data treatment – non-linear regression model

The 4-parameter logistic (4PL) model[1] was used to fit the dose-response curve. The 4PL model was used to determine the EC50 of the agonist, i.e., the concentration of an agonist that produces 50% of the maximal possible effect of that agonist. The model does not assume a standard slope of 1.0 but rather fits the Hill Slope (*HS*) from the data, and is thus called a variable slope model. If X is the logarithm of the concentration of the agonist, Y the response of the system to it, and Y_0 and Y_1 are the plateaus in the response at low and high values of X, respectively, the 4PL model is

$$Y = Y_0 + \frac{Y_1 - Y_0}{1 + 10^{(\log_{10}(EC50) - X) \times HS}} \quad \text{(S1)}$$

This model was used to fit all dose-response curves presented in this work. The obtained parameters for the fittings from the magnetic incubation study (Fig. 4 in the text) are presented in Table S1 below.

Table S1. Fitting parameters obtained with 4PL fitting of magnetic incubation curves

**Fitting parameters**	**Number of cycles**		
	60	120	180
EC50 (ng/mL)	1167	609	395
Hill Slope	1.2	1.2	1.3
R square	1.000	0.9996	0.9999

S4. MNP concentration dependence

Two MNP samples with concentrations of 0.1 mg/mL and 0.5 mg/mL were prepared and mixed with 6 μL of NS1 diluted in serum in six different concentrations. The assay was run using the above optimal conditions and the signal was acquired at the end. Figure S3 shows the dose-response curve for the two MNP concentrations. Lines are fits to the 4PL model described in Section S3.

Since the intensity of the optomagnetic signal depends on the number of MNPs in solution, to allow for comparison, the $V_2'(17\,\mathrm{Hz})$ values vs. NS1 concentrations were divided by the value obtained for the blank sample. The results show a considerable increase in the signal (~1.5-fold) when using 0.1 mg/mL MNPs. For lower MNP concentrations, a higher fraction of MNP is affected by the formation of specific nanoclusters. However, the signal to noise ratio also decreases for lower MNP concentrations. Overall, we find that the highest sensitivity is obtained for an MNP concentration of 0.1 mg/mL.

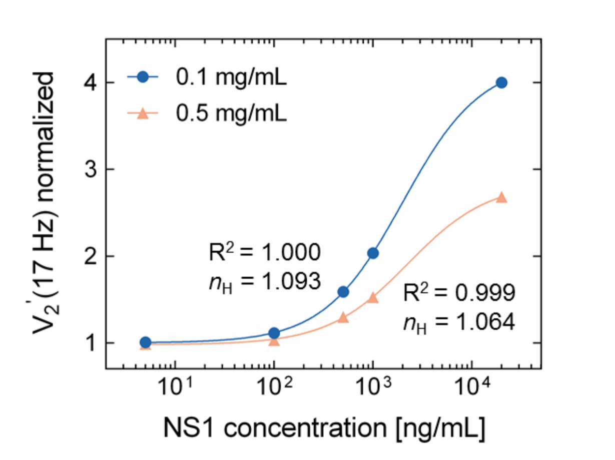

Fig. S3 Normalized values of $V_{2,}'(17\,\mathrm{Hz})$ vs. NS1 concentration in serum for MNP concentrations of 0.1 and 0.5 mg/mL. Values were normalized to signal obtained for the blank sample. The lines are fits to the 4PL model of Section S3.

References

1. Eckels, J. *et al.* Quality control, analysis and secure sharing of Luminex® immunoassay data using the open source LabKey Server platform. *BMC Bioinformatics* **14,** 145 (2013).
